# Inhibition of PD-1 Protects against TNBS-Induced Colitis via Alteration of Enteric Microbiota

**DOI:** 10.1155/2021/4192451

**Published:** 2021-01-07

**Authors:** Hao-ming Xu, You-lian Zhou, Jing Xu, Ying-fei Li, Chong Zhao, Hong-li Huang, Yan-lei Du, Jie He, Yong-jian Zhou, Yu-qiang Nie

**Affiliations:** Department of Gastroenterology and Hepatology, Guangzhou First People's Hospital, School of Medicine, South China University of Technology, Guangzhou, Guangdong, China

## Abstract

**Methods:**

Colitis was induced in mice using 2,4,6-trinitrobenzene-sulfonic acid (TNBS), and mice were subsequently treated with either a PD-1 inhibitor or 5-amino-salicylic acid (ASA) as a positive control. Body weight, disease activity index (DAI), colon length, and tissue damage were evaluated, and the enteric microbiota was profiled using high-throughput 16S rRNA sequencing of fecal samples from the experimental mice.

**Results:**

TNBS caused mice to experience IBD-like symptoms, which were attenuated by the PD-1 inhibitor, as indicated by a decrease in DAI scores (*p* = 0.0002). Furthermore, in this mouse model of IBD, PD-1 inhibition improved the alpha diversity as well as restored the beta diversity of the enteric microbiome. It also significantly enriched the abundance of short-chain fatty acid- (SCFA-) producing bacteria of the *Firmicutes* (*p* < 0.05) and *Bacteroidetes* (*p* < 0.05) phyla but depopulated *Proteobacteria* (*p* < 0.05).

**Conclusion:**

PD-1 inhibition can partly mitigate TNBS-induced colitis and restore the enteric microbiota by enriching the abundance of SCFA-producing bacteria.

## 1. Introduction

Inflammatory bowel disease (IBD) includes two chronic and nonspecific disease entities, ulcerative colitis and Crohn's disease [1], and clinically presents as abdominal pain, diarrhea, and bloody stool. Etiological studies have proposed genetic factors, a disproportionate immune response, an impaired mucoepithelial barrier, and dysbacteriosis as the putative pathogenic mechanisms for the development of IBD [2, 3].

Recent studies have suggested that aberrant immune activation against the enteric microbiota forms the pathogenic foundation of IBD [4, 5]. Experiments on enteric microbiota from IBD patients have revealed that a decreased abundance of the *Firmicutes* and *Bacteroidetes* phyla with an increased abundance of *Proteobacteria* is significantly correlated to the severity of IBD, and genera of the *Firmicutes* phylum, such as the *Clostridium*, *Ruminococcus*, *Lachnospira*, and *Roseburia* genera, as well as *Faecalibacterium prausnitzii* are reduced in IBD patients [6].

Immunocheckpoint blockade (ICB) has recently been popularized as a treatment for malignancies and other diseases. Clinically, not only does ICB possess the adverse effect of IBD-like enteritis itself, but during or after the course of ICB therapy, a significantly higher proportion of patients with IBD developed either common gastrointestinal adverse events (diarrhea, abdominal pain, etc.) compared to patients without IBD, or had a flare-up of their IBD symptoms [7–10], thus indicating the role of ICB in the progression of IBD.

However, there have not been many studies that have investigated the role of the PD-1/programmed death ligand 1 (PD-L1) axis in intestinal inflammation. Kanai et al. [11] found that PD-1 was highly expressed on T cells in the inflamed colon, while blockade of PD-L1 suppressed experimental colitis. In contrast, Scandiuzzi et al. [12] showed that PD-L1 expressed by the intestinal epithelium regulated intestinal inflammation by inhibiting innate immune cells. Moreover, Park et al. [13] suggested that the absence of PD-1 was protective against experimental colitis through the gut microbiota. Therefore, we aimed to investigate the pathogenic connection between the PD-1/PD-L1 pathway, the progression of IBD, and the enteric microbiota using a mouse model of 2,4,6-trinitrobenzene-sulfonic acid- (TNBS-) induced colitis.

## 2. Materials and Methods

### 2.1. Drugs and Reagents

The PD-1 inhibitor was purchased from TopAlliance Biosciences Inc. (Shanghai, China). TNBS and 5-amino-salicylic acid (ASA) were purchased from Sigma (St. Louis, MO, USA).

### 2.2. Experimental Animals, Induction of Colitis, and Treatment Regimen

Male BALB/c mice (23.1-28.4 g) were purchased from Guangdong Medical Laboratory Animal Center (GDMLAC; Certificate number SYXK 2013-0002, Foshan, China), and the experimental procedures were performed in accordance with the guidelines approved by the Animal Ethics Committee of GDMLAC. The protocols were approved by the Committee on the Ethics of Animal Experiments of GDMLAC. Mice were housed under a 12 h light/dark cycle with controlled temperature (24°C) and humidity (50-70%) and had access to food and water *ad libitum*.

All experimental mice in this study received a diet of SPF maintaining mice feed-1003 (provided by GDMLAC, production license No. (2019) 05073, executive standard: GB14924.3-2010). The ingredients included corn, soybean meal, flour, wheat flour, Peru fish meal, calcium hydrogen phosphate, stone powder, sodium chloride, vegetable oil, vitamins, amino acids, and minerals, and the food was sterilized via cobalt-60 irradiation.

The mice were acclimatized for seven days and randomly assigned to one of four groups: control (*n* = 12), TNBS (*n* = 10), TNBS+5-ASA (*n* = 12), and TNBS+PD-1 inhibitor (*n* = 12) groups (Supplementary Figure 1). Colitis was induced by rectal administration of TNBS as described by Morris et al. [14]. Briefly, the mice were fasted for 24 h, anesthetized with 0.4% pentobarbital sodium (0.1 mL/10 g) via intraperitoneal injection, and administered 25 mg TNBS per kg body weight in 50% ethanol. The mice in the control group were given an enema of only 50% ethanol. The TNBS-treated (0.1 mL/10 g) mice in the ASA or PD-1 treatment groups were, respectively, administered with either 5-ASA (150 mg/kg) through transgastric feeding or the PD-1 inhibitor (3 mg/kg) through intraperitoneal injections to avoid the acidic degradation of the PD-1 inhibitor in the stomach. The mice were monitored daily for weight loss, stool consistency, and the presence of blood on the anus or in the stool. Following the treatment regimen, stool samples were collected, and colonic tissues were resected after the mice were euthanized. The colon length was recorded, and the tissues were fixed overnight in 10% neutral buffered formalin.

### 2.3. Disease Activity Index

The disease activity index (DAI) was scored according to a modified version of a previously described method [15] and included the following parameters: (A) weight loss percentage (0: none, 1: 1-5%, 2: 5-10%, 3: 10-15%, and 4: >15%), (B) stool consistency (0: normal, 1: pasty and not sticking to the anus, 2: pasty and slightly sticking to the anus, 3: pasty and stuck to the anus, and 4: watery), and (C) rectal bleeding (0: hemoccult (-), 1: hemoccult (±), 2: hemoccult (+), 3: hemoccult (++), and 4: obvious blood in stool).

### 2.4. Histopathological Evaluation

The mice were euthanized under anesthesia, and the entire colon from cecum to anus was removed. The colon was opened longitudinally along the mesenteric border, and its contents were flushed with cold saline. The cleaned tissue samples were fixed in buffered formalin, embedded in paraffin, cut into 5 *μ*m sections, and stained with hematoxylin and eosin (H&E). The slides were histopathologically evaluated by two investigators blinded to the conditions and graded with the following parameters: (A) inflammation (0: no infiltration of inflammatory cells, 1: infiltration in the lamina propria, 2: infiltration into the submucosa, and 3: transmural infiltration), (B) ulceration (0: no ulceration, 1: one or two ulcers, 2: three or four ulcers, and 3: more than four ulcers), (C) mucosal hyperplasia (0: normal, 1: slightly thickened mucosa with minimal fibrosis, 2: mucosal thickening with fibrous hyperplasia, and 3: extensive mucosal thickening and fibrous hyperplasia or granulation), and (D) edema (0: none, 1: 0-30%, 2: 30-70%, and 3: >70%).

### 2.5. Fecal Sample Collection and Extraction of Genomic DNA

Approximately two or three pellets of fresh feces per mouse were collected in sterile plastic tubes and stored at -80°C. Total genomic DNA was extracted using the QIAamp DNA Stool Mini Kit (Qiagen, Dusseldorf, Germany) and quantified at 260 nm with the NanoDrop 2000 BioAnalyzer (Thermo Fisher Scientific, Inc., Waltham, MA, USA).

### 2.6. PCR Amplification and Illumina Sequencing

Bar-coded V4-515 forward 5′-GTGCCAGCMGCCGCGGTAA-3′ and V4-806 reverse 5′-GGACTACHVGGGTWTCTAAT-3′ primers were used to amplify the bacterial 16S rRNA V4 fragments. The Phusion High-Fidelity PCR Master Mix (New England Biolabs, Beverly, MA, USA) was used, and amplified products 400-450 bp in length were purified. Sequencing libraries were generated using the TruSeq DNA PCR-Free Sample Preparation Kit (Illumina, San Diego, CA, USA) according to the manufacturer's recommendations, and index codes were added. The library quality was assessed on the Qubit@ 2.0 Fluorometer (Thermo Scientific, Carlsbad, CA, USA) and Agilent Bioanalyzer 2100 system and sequenced using the Illumina HiSeq 2500 platform (Tianjin Novogene Bioinformatics Technology Co., Ltd.).

### 2.7. Data Analysis

SPSS statistical software was used to perform all analyses, and the data were expressed as the mean ± standard deviation (SD). *t*-tests and Wilcoxon signed-rank tests were used to compare two groups when appropriate, and one-way analysis of variance (ANOVA) with Tukey's tests was used for comparisons of multiple groups. A *p* value less than 0.05 was considered statistically significant.

## 3. Results

### 3.1. Intestinal Inflammation in TNBS-Induced Colitis Is Partially Alleviated by Blocking PD-1/PD-L1

For generating the experimental models, 10 mice were treated by rectal administration of TNBS, all of which showed significant weight loss and higher DAI scores associated with diarrhea, rectal bleeding, and shorter colon lengths compared to the control mice, indicating the successful establishment of colitis as a mouse model of IBD, with a success rate of 100% (1 of 10 died on the seventh day due to severe diarrhea and rectal bleeding).

**Figure 1:**
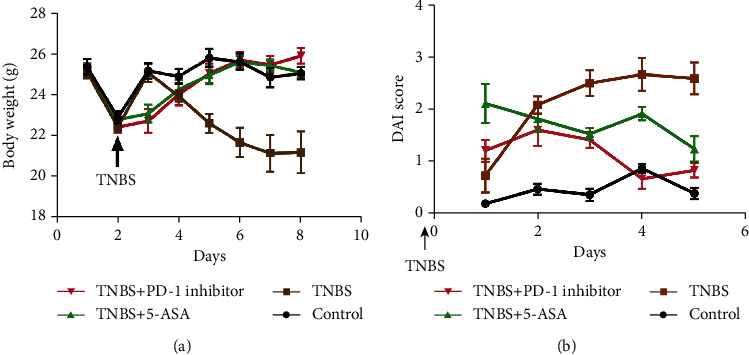
Inhibition of programmed cell death protein 1 (PD-1) improved the physiological indices in mice with TNBS colitis. (a) Body weight and (b) disease activity index (DAI) in the various treatment groups. Data is expressed as the mean ± standard deviation (SD), ^∗^*p* < 0.05.

**Figure 2:**
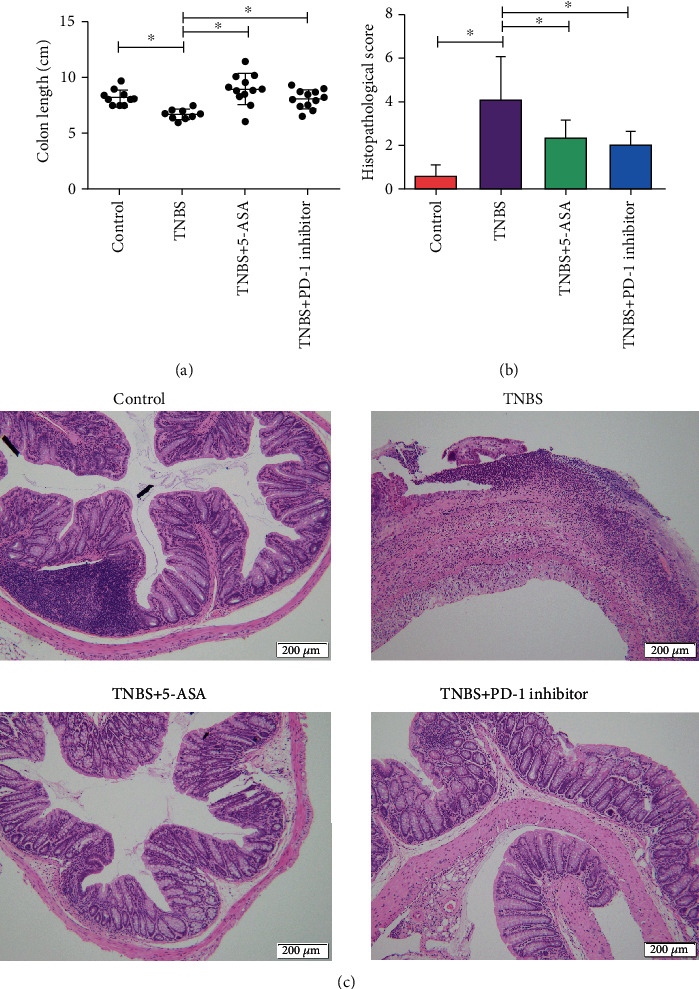
Inhibition of programmed cell death protein 1 (PD-1) restored macroscopic and histopathological damage caused by TNBS-induced colitis. Representative pictures showing the (a) colon length and (b, c) histopathological appearance of colonic tissue (scale bar = 200 *μ*m). Data are expressed as the mean ± standard deviation (SD), ^∗^*p* < 0.05.

Administration of either the PD-1 inhibitor or 5-ASA (as positive control) prevented the mice with IBD-like colitis from weight loss (Figure 1(a)) and improved their DAI scores (*p* = 0.0002) compared to the untreated mice with colitis (Figure 1(b)). In addition, treatment with the PD-1 inhibitor mitigated inflammation along the intestinal mucosa in mice with TNBS-induced colitis as indicated by a decrease in cell infiltration and reorganization of the intestinal villi structure (Figures 2(b) and 2(c)).

### 3.2. PD-1 Inhibition Increases Enteric Microbiota Diversity in Mice with Colitis

**Figure 3:**
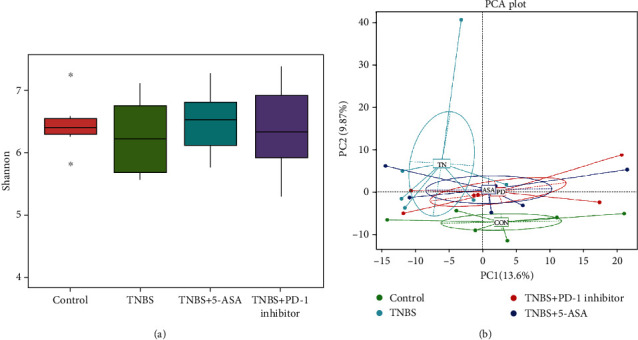
Inhibition of PD-1 increased the alpha diversity and restored the beta diversity of the gut microbiota in mice with TBNS colitis. (a) Shannon index and (b) principal component analysis (PCA). Each dot represents one fecal sample.

The Shannon index was used to estimate the alpha diversity, a measure of taxa richness, and evenness within a sample. Both the PD-1 inhibitor and 5-ASA increased the low alpha diversity after TNBS-induced colitis (Figure 3(a)). In addition, principal component analysis (PCA) was used to evaluate the beta diversity by comparing the similarity in microbial communities between samples. The PD-1 inhibitor reversed the changes in the enteric microbiota caused by TNBS (Figure 3(b)).

### 3.3. PD-1 Inhibitor Restores the Gut Microbiota and Increases the Abundance of Short-Chain Fatty Acid-Producing Bacteria

The relationship between gut microbial composition and IBD has been widely investigated, and IBD patients show a significant decline in the diversity of their intestinal microbiome along with a decrease in the abundance of *Firmicutes* and an increase in the abundance of *Proteobacteria*, which are designated as signatures of IBD [4].

**Figure 4:**
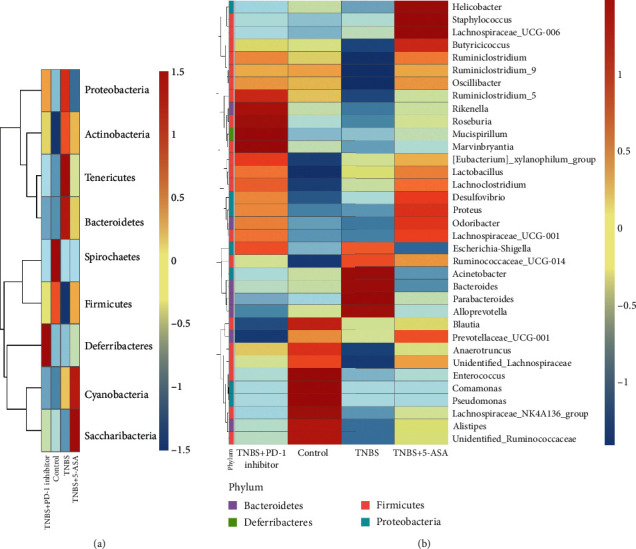
Cluster heat map analysis of the (a) top nine predictive operational taxonomic units (OTUs) at the phylum level and (b) top 35 predictive OTUs at the genus level. Blue indicates a negative correlation, and red indicates a positive correlation. The *X*-axis indicates the sample information, and the *Y*-axis contains the annotation information for the species. The tree on the left indicates species clustering.

**Figure 5:**
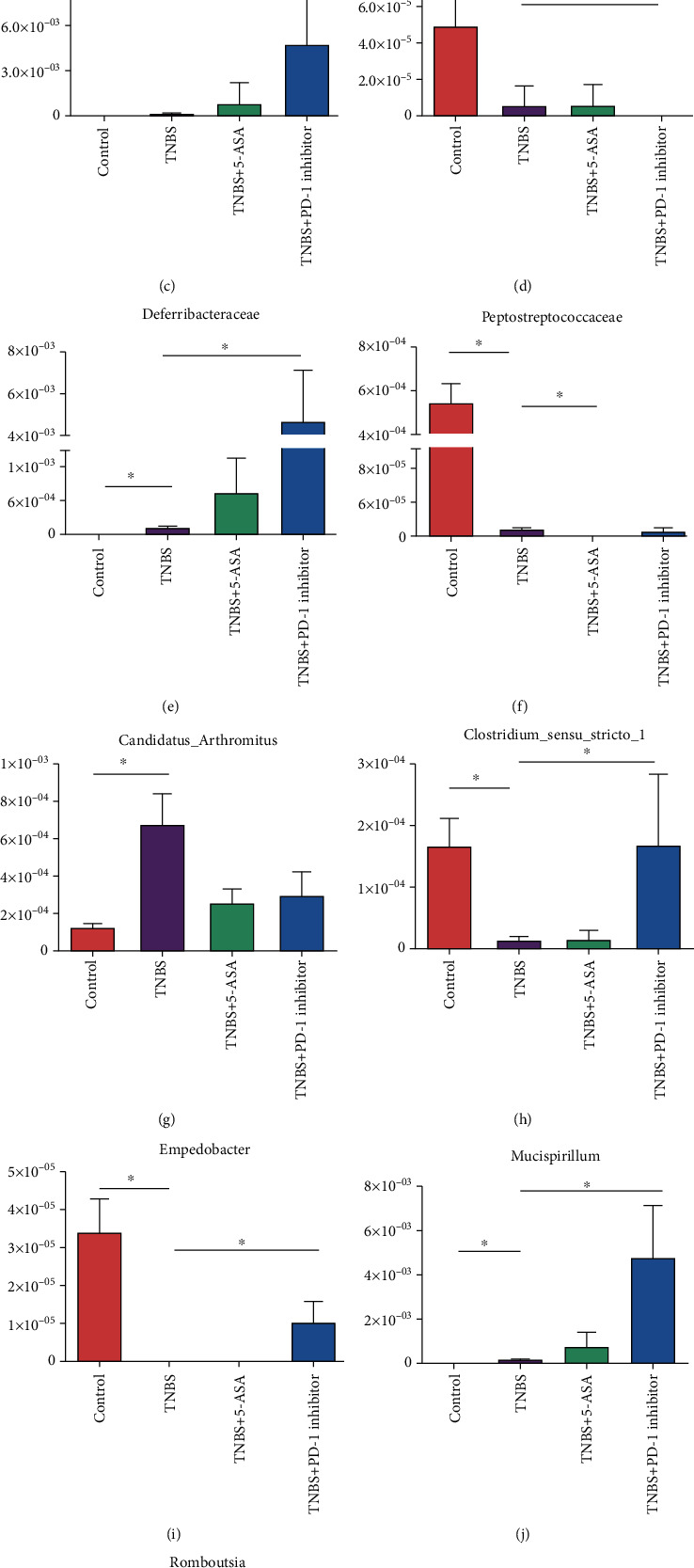
Differential bacterial communities identified by Metastats at the level of (a) phylum, (b) class, (c, d) order, (e, f) family, and (g–l) genus; ^∗^*p* < 0.05.

**Figure 6:**
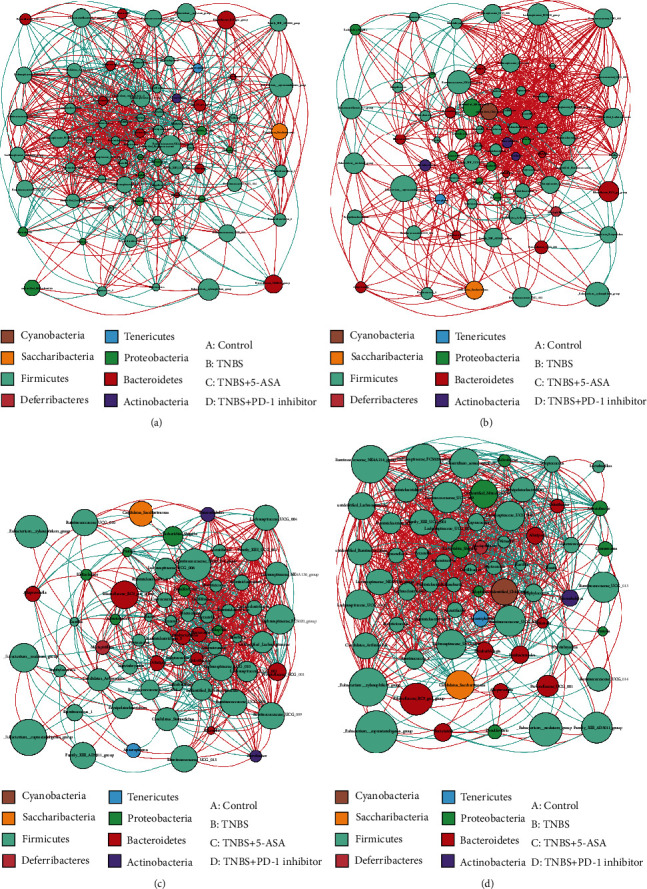
Network maps showing the interactions of the operational taxonomic units (OTUs) among all the samples at the genus level. Each point represents one genus: (a) control group; (b) TNBS group; (c) TNBS+5-ASA group; (d) TNBS+PD-1 inhibitor group.

The heat maps for the predictive operational taxonomic units (OTUs) were plotted according to the species annotations and abundance at the phylum and genus levels, and a cluster analysis was performed (Figure 4). These results suggested that the inhibition of PD-1 increased the abundance of *Butyricicoccus*, *Ruminiclostridium*, *Ruminiclostridium 9*, *Oscillibacter*, *Ruminiclostridb-5*, *Rikenella*, *Roseburia*, *Anaerotruncus*, *Lachnospiraceae bacterium NK4A136*, and an unidentified *Lachnospiraceae* but decreased that of *Acinetobacter*, *Bacteroides*, *Parabacteroides*, and *Alloprevotella*. Metastats was used to analyze the relative abundance of bacteria in each group at different levels. Inhibition of PD-1 increased the abundance of *Deferribacteres*, *Clostridium_sensu_stricto_1*, *Empedobacter*, and *Mucispirillum* and decreased that of *Rhizobiales*, *Candidatus_Arthromitus*, and *Turicibacter* (Figure 5). As shown in the network map in Figure 6, the mice in the control groups possessed *Firmicutes*, *Bacteroidetes*, and *Proteobacteria* as the core genera, while TNBS-induced colitis resulted in the enrichment of *Actinobacteria* and *Cyanobacteria*. After treatment with the PD-1 inhibitor, the relative abundance of *Firmicutes* and *Bacteroidetes* was restored in the mice with TNBS-induced colitis to levels comparable to the control groups. Taken together, inhibiting PD-1/PD-L1 signaling restored the enteric microbiota dysbiosis in mice with colitis by enriching the abundance of SCFA-producing bacteria as well as mucosal immune-related bacteria.

## 4. Discussion

The pathogenesis of IBD in genetically susceptible hosts has been proposed to begin with a breakdown of the intestinal epithelial barrier, followed by a disproportionate immune response to the enteric microbiota, which results in a loss of intestinal homeostasis [16]. Such dysbiosis in the intestinal symbionts of IBD patients can be indicated by the low-level alpha diversity [17] and manifests as a decrease and an increase in the abundance of *Firmicutes* and *Proteobacteria*, respectively [18]. A study of the enteric microbiota in gnotobiotic mice under an inflammatory microenvironment [19] revealed that the enteric microbiota potentiates the formation and maturation of CD4^+^ T cells, and the severely damaged intestinal mucoepithelial barrier in IBD patients exposes the gut microbial antigens to the immune cells, which elicit an excessive immune response. Furthermore, various metabolites of the intestinal bacteria, such as SCFAs, can increase the number of immunosuppressive T_reg_ cells in the laminae propria mucosae [20, 21] and promote mucin secretion as well as strengthen the epithelial barrier. Studies show that maintaining a minimal concentration of SCFAs in the intestine is beneficial to the repair and regeneration of the mucosal barrier, whereas chronic lack of SCFAs can thin the intestinal mucosa and weaken the integrity of the intestinal mucosal barrier, resulting in the translocation of pathogenic bacteria and increased exposure to antigenic substances [22–26].

Since the PD-1/PD-L1 pathway regulates T cell activation and immune tolerance and has been a potent therapeutic target in autoimmune diseases [27], recent studies began to focus on the role of the PD-1/PD-L1 signaling axis in the pathogenesis of IBD and possible novel therapeutic strategies. However, very few studies made significant progress. We used a common murine model of chronic IBD that involved rectal administration of TNBS [14, 28, 29], and we administered a PD-1 inhibitor, or 5-ASA as a positive control, to determine the pathogenic association of the PD-1/PD-L1 pathway with IBD. Our findings revealed that inhibition of PD-1 can significantly improve the physiological status of mice with colitis based on the evaluation of body weight along with DAI scores, and it also reduced both macroscopic and microscopic colonic lesions.

Past studies have focused more on the immunological regulation of the PD-1/PD-L1 pathway. However, Kawamoto et al. [30] investigated the effect of the PD-1/PD-L1 pathway on the enteric microbiota and reported a significant decrease in the relative abundance of *P. aeruginosa* and *Bifidobacteria* in the intestinal microbiota of PD-1^−/−^ mice, whereas Vetizou et al. [31] were followed to show that the introduction of *Bacteroides fragilis* alleviated colitis caused by cytotoxic T-lymphocyte-associated protein 4 (CTLA-4) inhibition. Based on the knowledge from these previous studies and with our experimental model, we then analyzed the diversity and abundance of the enteric microbiota in the different experimental groups and upheld that PD-1 inhibition increased microbial diversity and altered the abundance of various phyla and genera, especially bacteria that produced SCFAs.

*Lachnospiraceae*, *Rikenellaceae*, *Ruminococcaceae*, *Butyricicoccus*, and *Roseburia* are important SCFA-producing intestinal symbiotic bacteria, which ferment dietary fiber into acetic acid, propionic acid, and butyric acid [32]. More specifically, Eeckhaut et al. [33] revealed a lower abundance of *Butyricicoccus* in IBD patients, while the introduction of *Butyricicoccus* in mice with TNBS-induced colitis reduced levels of proinflammatory factors including myeloperoxidase (MPO), tumor necrosis factor alpha (TNF-*α*), and interleukin- (IL-) 12 and improved intestinal mucosal barrier function. Several other studies [34–37] have also reported that IBD patients possessed a lower abundance of *Lachnospiraceae*, a bacteria that can protect the intestinal tract through the production of butyrate and decolonize *C. difficile* during pseudomembranous colitis. More importantly, cancer patients with a higher abundance of *Rikenellaceae* undergoing anti-CTL4 therapy were less likely to suffer from CTLA-4-associated enteritis [31]. The abundance of the butyrate-producing actinomycete *Roseburia* [38] is decreased in ulcerative colitis patients, and another butyrate-producing bacteria, *P. sphaeroides*, is associated with disease activity in patients with Crohn's disease [39, 40]. *Ruminococcaceae* and *Lachnospiraceae*, the two most abundant bacteria of the *Clostridium spp*, decompose various fibrous polysaccharides and are associated with the PD-1/PD-L1 pathway [41]. The SCFA-producing bacteria also promote protein synthesis that can affect the intestinal immune response [42]. Schaffler et al. [43] found that an increased abundance of *Anaerotruncus* enhanced the therapeutic effect against Crohn's disease. Taken together, SCFA-producing enteric bacteria not only manufacture SCFAs as the main source of energy for the intestinal microbes but also help to maintain intestinal microbial homeostasis by inhibiting the growth of pathogenic bacteria, promoting colonic mucus secretion, and enhancing mucosal barrier function [44]. Butyrate enemas have been used to manage mucosal inflammation in IBD, highlighting the therapeutic potential of SCFAs for the treatment of IBD [45, 46].

An alteration in the abundance of certain enteric microbes has been associated with intestinal mucosal immune regulation. For example, *Bacteroides acidifaciens* induces the formation of germinal centers and regulates immunoglobulin A (IgA) and IgB-producing plasma cells [47], while *Clostridium leptum* induces immunotolerant dendritic cells and T_reg_ cells [48–50]. IgA is the major antibody on the surface of the intestinal mucosa which can penetrate the intestinal lumen via the multi-immunoglobulin receptor (pIgR) present on intestinal epithelial cells to mediate intestinal mucosal immunity [51, 52]. Other enteric microbes such as *Mucispirillum* and *Candidatus_Arthromitus* also possess a strong correlation with the production and secretion of T cell-dependent IgA [53, 54]. Importantly, the abundance of *Mucispirillum* increased significantly after inhibition of PD-1, and therefore, it may serve as a potential marker for the inhibition of this pathway [55, 56].

Although the cross-talk between the PD-1/PD-L1 pathway, gut microbiota, and intestinal mucosal immunity has been investigated in detail [57, 58], the exact involvement of the PD-1/PD-L1 axis in the pathogenesis of IBD remains elusive. PD-1 inhibitors significantly improved the outcome of TNBS-induced colitis by restoring the intestinal microbiome. However, the dosage, withdrawal criteria, and other immunological effects of PD-1 inhibition require additional studies.

## 5. Conclusion

PD-1 inhibition can partly alleviate TNBS-induced colitis and restore the gut microbiota by increasing the abundance of SCFA-producing bacteria, but the exact effects and mechanisms require additional study.

## Data Availability

The dataset used and analyzed during the current study is available from the corresponding authors on reasonable request.

## Supplementary Materials

Supplementary Materials Supplementary Figure 1: process of TNBS-induced colitis model and solvent control. Preparation of TNBS ethanol solution: in 10 mL of 50 mg/mL TNBS solution, add 10 mL absolute ethanol and mix well. Preparation of 50% ethanol solution: in 2 mL of absolute ethanol, add 2 mL sterile water for injection and mix well. Step 1 (fasting): all mice were fasted for 24 hours. Step 2 (anesthesia): mice were anesthetized by intraperitoneal injection of pentobarbital sodium at the dose of 40 mg/kg and 0.1 mL/10 g body weight. Step 3 (enema): 25 mg/L TNBS ethanol solution (model group)/50% ethanol solution (control group) was aspirated by syringe. The soft rubber tube connected with the syringe was gently inserted approximately 3 cm into the mouse through the anus, and the contents of the syringe were slowly injected into the intestinal cavity of mice. Step 4 (inversion): after administration, the plastic tube was slowly removed and the tail of the mouse was lifted. The mouse was kept inverted for 1 min.
