# Supplementary figures and images for: Inhibition of PD-1 Protects against TNBS-Induced Colitis via Alteration of Enteric Microbiota
Source: Biomed Res Int. 2021 Jan 7;2021:4192451. doi: 10.1155/2021/4192451 (PMC7810563; doi:10.1155/2021/4192451)

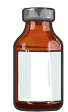

pentobarbital sodium solution

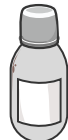

50% ethanol solution

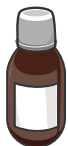

TNBS ethanol solution

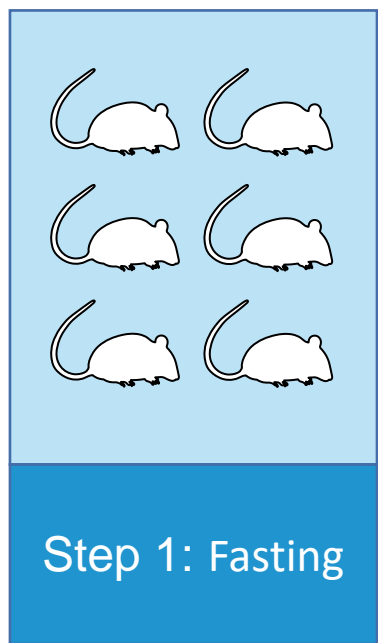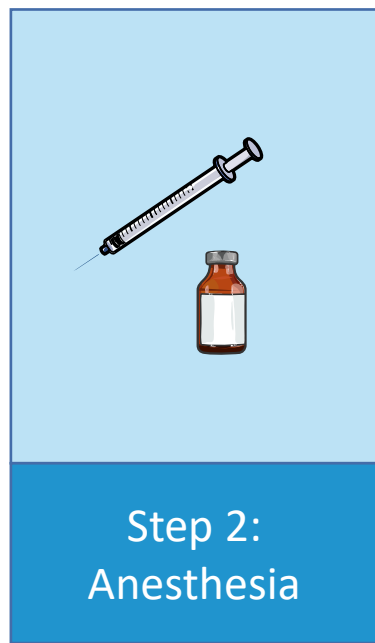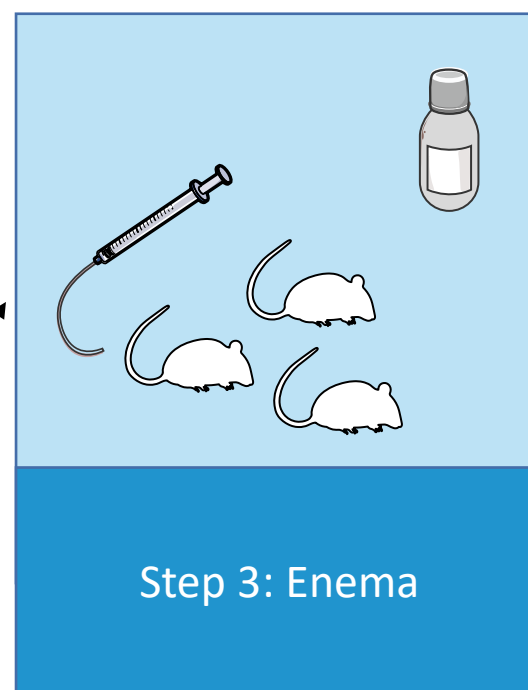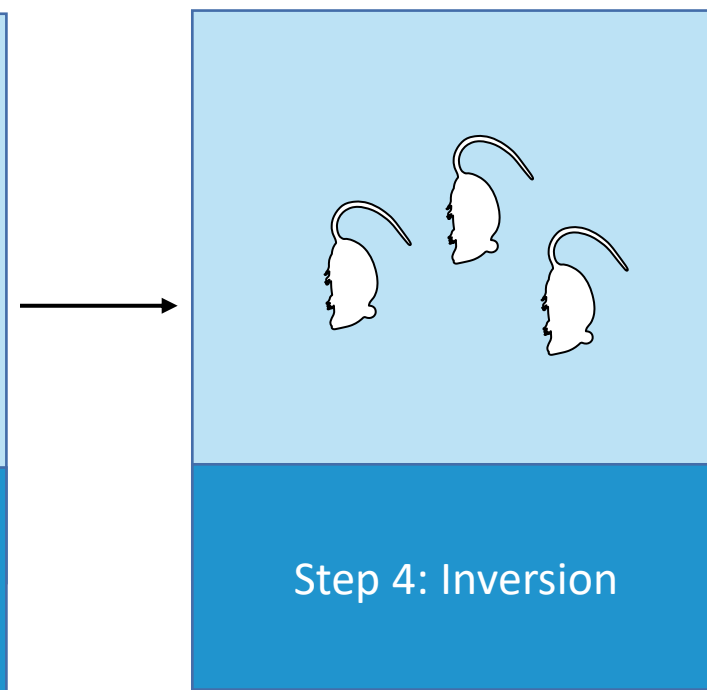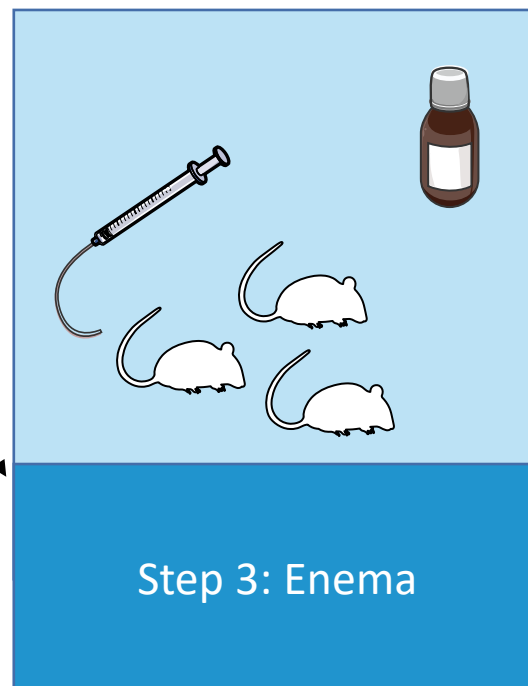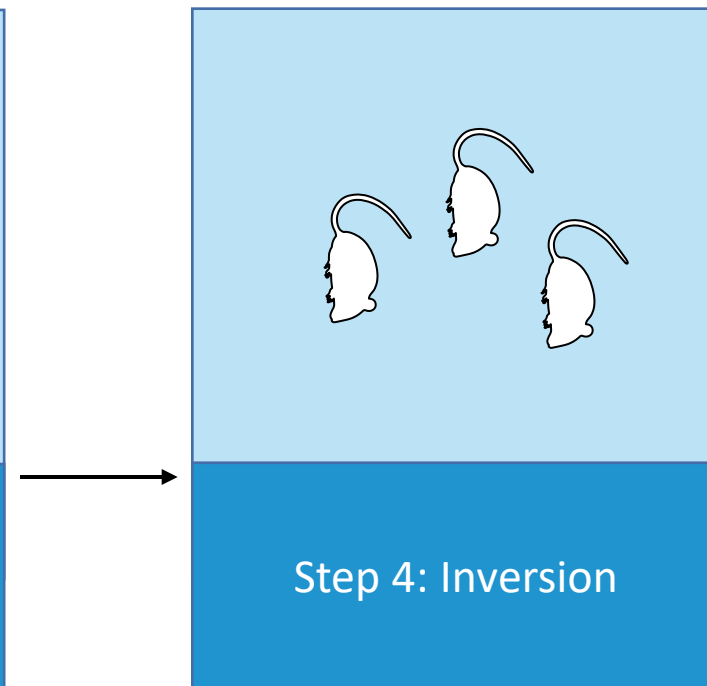

Supplement: Supplementary Materials — Supplementary Figure 1: process of TNBS-induced colitis model and solvent control. Preparation of TNBS ethanol solution: in 10 mL of 50 mg/mL TNBS solution, add 10 mL absolute ethanol and mix well. Preparation of 50% ethanol solution: in 2 mL of absolute ethanol, add 2 mL sterile water for injection and mix well. Step 1 (fasting): all mice were fasted for 24 hours. Step 2 (anesthesia): mice were anesthetized by intraperitoneal injection of pentobarbital sodium at the dose of 40 mg/kg and 0.1 mL/10 g body weight. Step 3 (enema): 25 mg/L TNBS ethanol solution (model group)/50% ethanol solution (control group) was aspirated by syringe. The soft rubber tube connected with the syringe was gently inserted approximately 3 cm into the mouse through the anus, and the contents of the syringe were slowly injected into the intestinal cavity of mice. Step 4 (inversion): after administration, the plastic tube was slowly removed and the tail of the mouse was lifted. The mouse was kept inverted for 1 min. [file 4192451.f1.pdf]
